# Carbon Coated Boron Nitride Nanosheets for Polymer Nanocomposites with Enhanced Dielectric Performance

**DOI:** 10.3390/ma10070741

**Published:** 2017-07-03

**Authors:** Minhao Yang, Hang Zhao, Delong He, Chaohe Hu, Haowei Chen, Jinbo Bai

**Affiliations:** 1 Laboratoire de Mécanique des Sols, Structures et Matériaux, CNRS UMR 8579, Centrale-Supélec, Université Paris-Saclay, Grande Voie des Vignes, 92290 Châtenay-Malabry, France; minhao.yang@ecp.fr (M.Y.); jmlhch@sina.cn (C.H.); 2 State Key Lab Incubation Base of Photoelectric Technology and Functional Materials, International Collaborative Center on Photoelectric Technology and Nano Functional Materials, Northwest University, Xi’an 710069, China; zhaohang_ecp@126.com (H.Z.); chenhaowei2005@126.com (H.C.)

**Keywords:** boron nitride nanosheets, chemical vapor deposition, hybrids, PVDF nanocomposites, dielectric constant

## Abstract

Carbon coated boron nitride nanosheets (BNNSs@C) hybrids with different carbon contents were synthesized by a chemical vapor deposition (CVD) method. The content of carbon in as-obtained BNNSs@C hybrids could be precisely adjusted from 2.50% to 22.62% by controlling the carbon deposition time during the CVD procedure. Afterward, the BNNSs@C hybrids were subsequently incorporated into the polyvinylidene fluoride (PVDF) matrix to fabricate the BNNSs@C/PVDF nanocomposites through a combination of solution and melting blending methods. The dielectric properties of the as-obtained BNNSs@C/PVDF nanocomposites could be accurately tuned by adjusting the carbon content. The resultant nanocomposites could afford a high dielectric constant about 39 (10^3^ Hz) at BNNSs@C hybrids loading of 30 vol %, which is 4.8 times larger than that of pristine BNNSs-filled ones at the same filler loading, and 3.5 times higher than that of pure PVDF matrix. The largely enhanced dielectric performance could be ascribed to the improved interfacial polarizations of BNNSs/carbon and carbon/PVDF interfaces. The approach reported here offers an effective and alternative method to fabricate high-performance dielectric nanocomposites, which could be potentially applied to the embedded capacitors with high dielectric performance.

## 1. Introduction

The tremendous advances in flexible electronic devices, pulsed-power electronic devices, and electric power systems have stimulated enormous research interests in the field of high dielectric constant materials [1,2,3,4,5,6,7,8,9,10]. Polymer materials with a high dielectric constant have exhibited prominent potential for high-performance capacitors in the past decades, owing to their inherent advantages in mechanical flexibility, processing ease, and low cost [11,12,13,14]. Nevertheless, the intrinsic low dielectric constant values (ca. <10) of polymer materials restricts their developments in polymer based novel dielectrics [15,16]. Therefore, the key issue is to substantially improve the dielectric constant of polymer materials, meanwhile retaining their other excellent properties, such as flexibility, low dielectric loss, and high breakdown strength. The nanocomposites technology provides a promising strategy to achieve a high dielectric constant by incorporating the conductive fillers or inorganic ceramics into the polymer matrix. According to the fillers used, these nanocomposites could be divided into two categories. The first type of nanocomposites (percolative nanocomposites) is obtained by incorporating the conductive fillers into the polymer matrix, where a giant dielectric constant is achieved as the volume fraction of the conductive fillers approaches to the vicinity of the percolation threshold (*f_c_*) [17,18,19,20]. However, a simultaneously obtained dielectric loss is always unavoidable. The other type of nanocomposites is prepared by homogeneously dispersing the inorganic ceramic fillers with a high dielectric constant into a polymer matrix [21,22,23,24,25]. Whereas a considerable dielectric constant is always accompanied with a high filler loading (>50 vol %), which would severely deteriorate the flexibility and electrical breakdown strength of polymer matrix. Many efforts have been made to reduce the filler loading while retaining a high dielectric constant over the last few years [26,27,28,29].

Recently, encapsulating ceramic fillers with conductive layers has drawn tremendous interests to promote the enhancement of dielectric constant for inorganic/polymer nanocomposites [30,31,32,33]. According to the conductive layer used, these encapsulating approaches could be mainly classified into two categories. The first type of coating process is obtained by depositing metal particles on the surface of ceramic fillers. For instance, Luo and his co-workers reported that the agglomeration of Ag nanoparticles in polyvinylidene fluoride (PVDF) matrix could be suppressed by utilizing the Ag-deposited BaTiO_3_ (BT-Ag) hybrids, which endows the nanocomposite with a high dielectric constant about 94.3 at 43.4 vol % filler loading [30]. The similar CaCu_3_Ti_4_O_12_ and Ag hybrids (CCTO@Ag) were also synthesized and their corresponding PI nanocomposites could afford a high dielectric constant about 103 at 3 vol % filler loading [31]. The other type of conductive layer is carbon layer. The core–shell structured ceramic/carbon hybrids have already exhibited their potential advantages in enhancing the dielectric constant of nanocomposites. For example, Feng et al. prepared core–shell structured BaTiO_3_@carbon (BT@C) hybrids by the chemical vapor deposition (CVD) method and these hybrids were incorporated into the P(VDF-HFP) to fabricate BT@C/P(VDF-HFP) nanocomposites, which endows the nanocomposites with a giant dielectric constant about 1044 at 30 vol % filler loading [32]. The flower-like TiO_2_/carbon (TiO_2_/C) hybrids were also fabricated by a solvothermal method and they were subsequently adopted as fillers to prepare P(VDF-HFP) nanocomposites, which affords a dielectric constant about 330.6 at 20 vol % filler loading [33]. All these previous studies have confirmed that the core–shell structured hybrids with a conductive layer could endow the nanocomposites with an excellent dielectric property.

The incorporation of hexagonal boron nitride nanosheets (BNNSs) into polymer matrix have been widely reported in last few years because of its high breakdown strength (ca. 800 KV/mm), high thermal conductivity, extraordinary anti-oxidation stability, and excellent mechanical properties [8,9,34]. It has been well demonstrated that incorporation of BNNSs into polymer matrix could inhibit the mobility of the charge carrier, thus resulting in a dramatic decrease of dielectric loss. However, the applications of polymer nanocomposites incorporated with BNNSs in embedded capacitors are greatly hindered by the intrinsic low dielectric constant of BNNSs (ca. 3–4). In order to further improve the dielectric constant of BNNSs incorporated polymer nanocomposites, as discussed in above parts, the combination of the conductive layer with BNNSs provides a promising strategy to enhance the dielectric constant [34]. Our previous research has demonstrated that the existence of BNNSs could be used to adjust the conductive network of carbon nanotubes (CNTs)/PVDF nanocomposites [6]. However, the huge dielectric loss was also obtained due to the occurrence of percolation behavior. Therefore, it is imperative to rationally design an appropriate interaction between BNNSs and carbon conductive layer. Fu et al. immobilized the graphene oxide (GO) on the surface of BNNSs by electrostatic self-assembly and subsequently introduced these hybrids into epoxy accompanied with chemical reduction [34]. The reduced graphene oxide (rGO) sheets are well separated from each other even at a high hybrids loading. The enhancement of dielectric constant is accompanied with a low dielectric loss, which exhibits a competent dielectric performance. The low dielectric loss could be ascribed to the insulating network of BNNSs to inhibit the mobility of charge carrier and well-separated rGO sheets by immobilization. However, the enhancement degree of dielectric constant is limited due to the limitation of the quantity of graphene sheets immobilized on the BNNSs surface.

Many previous studies demonstrated that the similar hexagonal structure of BNNSs and graphene layer favored the carbon deposition on the surface of BNNSs [35]. In this study, the BNNSs@carbon (BNNSs@C) hybrids with different carbon contents were synthesized by a CVD method because of its simplicity and controllability [36,37,38,39,40]. The carbon content in BNNSs@C hybrids could be precisely controlled by adjusting the carbon deposition time during the CVD procedure. The BNNSs@C hybrids were subsequently filled into the PVDF matrix by a combination of solution and melting blending processes. The dielectric performances of the as-obtained BNNSs@C/PVDF nanocomposites could be precisely controlled by tuning the carbon content in BNNSs@C hybrids. These results indicate that the dielectric performance of BNNSs@C/PVDF nanocomposites could be largely enhanced after depositing the carbon layer on the surface of BNNSs. The design concept here provides a new thought for the preparation of dielectric materials with the excellent dielectric performance, which makes them a prominent potential candidate in embedded capacitor industry.

## 2. Experimental

### 2.1. Synthesis of BNNSs@C Hybrids Fillers

The BNNSs@C hybrids were fabricated by the CVD method using C_2_H_2_ as the carbon source. The BNNSs with a lateral size about 0.5–2 µm were supplied by Merck Millipore (Darmstadt, Germany). In brief, a certain amount of pristine BNNSs was homogeneously dispersed on a quartz plate to serve as the substrate for carbon deposition. The furnace was heated to 850 °C under an Ar (1000 s.c.c.m.) atmosphere and annealed for 5 min with a certain amount of H_2_ to reduce the surface oxygen functional group of BNNSs. A small amount of C_2_H_2_ (40 s.c.c.m.) was subsequently introduced into the reaction tube at ambient pressure. After the synthesis, the reactor was cooled down to the room temperature under the Ar atmosphere (1000 s.c.c.m.). The quartz plate with the BNNSs@C hybrids was carefully taken out of the reactor. The resultant powder after carbon deposition was separately collected using a thin cutting blade. The time for the carbon deposition was fixed in the range of 5–45 min.

### 2.2. Preparation of BNNSs/PVDF and BNNSs@C/PVDF Nanocomposites

The BNNSs@C/PVDF nanocomposites with various BNNSs@C filler loadings (*f_BNNSs@C_*) were prepared by a combination of solution casting and extrusion–injection methods in order to achieve a good and stable dispersion state of BNNSs@C hybrids in PVDF matrix. Briefly, a desired amount of BNNSs@C hybrids was ultrasonically dispersed into *N*, *N*-dimethylformamide (DMF) solvent for 2 h. The PVDF powders (Kynar 761, Arkema Group, Colombes, France) were subsequently introduced into the suspension of BNNSs@C hybrids and the mixture was magnetically stirred at 70 °C for 2 h. Afterward, the temperature of mixture solution was increased up to 100 °C and held for 1 h to remove the majority of DMF solvent. The residual solvent was further removed by drying the pre-mixture in a vacuum oven at 80 °C for 12 h. The as-obtained BNNSs@C/PVDF mixture was subsequently melt-blended under Ar protective atmosphere by a two-screw micro-extruder with a speed of 90 rpm at 220 °C for 30 min. Finally, the slabs with a thickness of 1.5 mm were prepared by injection molding of the nanocomposites. The obtained *f_BNNSs@C_* in the BNNSs@C/PVDF nanocomposites varied from 0 to 30 vol %. The BNNSs/PVDF nanocomposites were prepared by the similar procedures, where *f_BNNSs_* represents the filler loading of pristine BNNSs.

### 2.3. Characterizations

Morphological characterizations of the BNNSs@C hybrids were performed by TEM using an FEI Titan instrument equipped with a high-angle annular dark-field (HAADF) detector and an aberration-corrected condenser operating at 200 kV. The elemental mapping was carried out using scanning TEM-energy dispersive X-ray spectroscopy (STEM-EDX). The observation of the freeze-fractured cross-section surfaces of nanocomposites was conducted by the SEM (LEO Gemini 1530, Zeiss, Oberkochen, Germany) operated at 5.0 kV. X-ray diffraction (XRD) patterns were measured on an XRD detector D2 PHASER (Bruker, Karlsruhe, Germany) with X-Flash 430 (Bruker, Karlsruhe, Germany)). Raman spectroscopy was performed with a Jobin Yvon LabRAM spectrometer (Horiba Scientific, Villeneuve d’Ascq, France). Thermogravimetric analysis (TGA, STA 449 F3, Netzsch, Selb, Germany) was used to evaluate the mass fraction of carbon. The furnace of TGA instrument with the powders was heated from 50 to 800 °C at a rate of 5 °C min^−1^ under N_2_ (20 s.c.c.m.) and O_2_ (20 s.c.c.m.) atmosphere. For dielectric measurements, electrodes were painted with silver paste on both sides of the samples and then the dielectric properties were characterized by a Novocontrol Alpha-A high performance frequency analyzer (Novocontrol Technologies, Montabaur, Germany) at room temperature.

## 3. Results and Discussion

**Figure 1:**
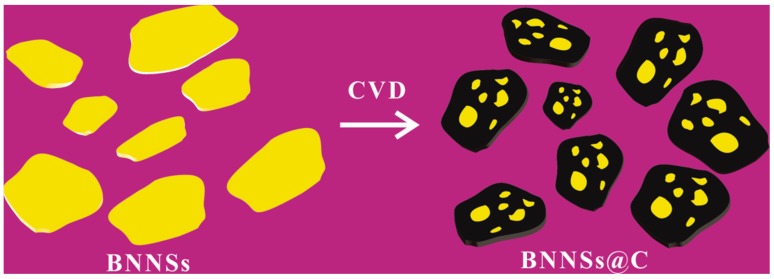
Schematic illustration of the synthesis procedure of BNNSs@C hybrids.

As shown schematically in Figure 1, the BNNSs@C hybrids were synthesized by the CVD method using C_2_H_2_ as the carbon source and Ar as the carrier gas at 850 °C for various reaction time. An appropriate amount of H_2_ was introduced to reduce the surface oxygen functional groups of BNNSs at the elevated temperature, which could eliminate their influences on the ultimate dielectric performance of polymer nanocomposites. The BNNSs have been extensively utilized as the substrate for the graphene growth due to their similar lattice parameters. As demonstrated in our previous work [5], when the temperature for carbon deposition is below 850 °C, the deposited carbon interlayer exhibits disorder graphene layers. Considering the merits of the conductive carbon layer on the final dielectric performance of nanocomposites, the temperature for carbon deposition in this work was fixed at 850 °C. For convenience and simplicity, the BNNSs@C hybrids with the different carbon deposition time are given by BNNSs@C-x, where x represents the carbon deposition time (min).

**Figure 2:**
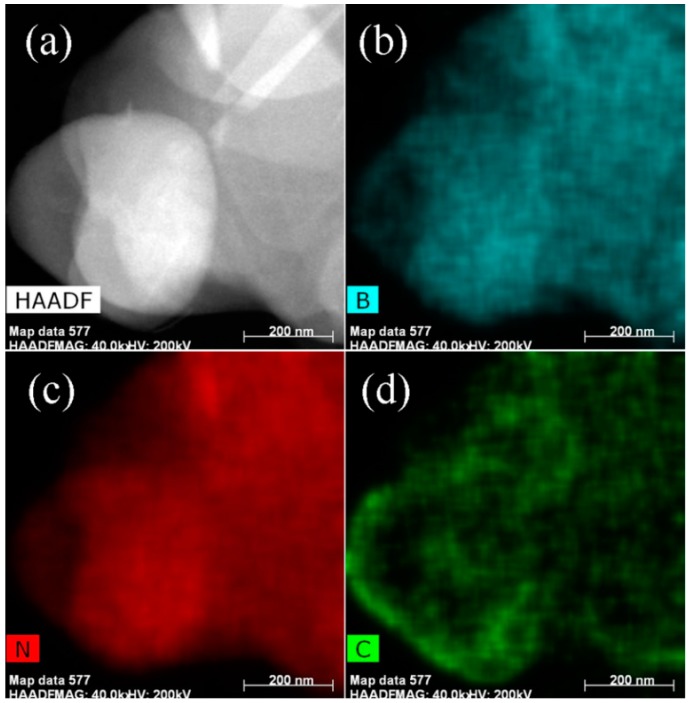
(**a**) HAADF-STEM image of the BNNSs@C-15 hybrids; (**b**–**d**) EDX elemental maps of B, N, and C, respectively.

STEM measurements were conducted to identify the morphology of the BNNSs@C-15 hybrids. Figure 2a shows the HAADF-STEM image of the BNNSs@C hybrids with a mean lateral size about 0.5–2 µm. The corresponding EDX elemental maps of BNNSs@C-15 hybrids are displayed in Figure 2b–d. The elements B, N, and C are denoted in aqua green, red, and green, respectively. The BNNSs are uniformly covered by a continuous carbon layer for the most part. However, some naked BNNSs portions could be observed due to the existence of defects. The STEM-EDX technique employed in this work could map out the elemental distribution at a nanometer-scale spatial resolution and provide a clear vision of the BNNSs@C hybrids.

**Figure 3:**
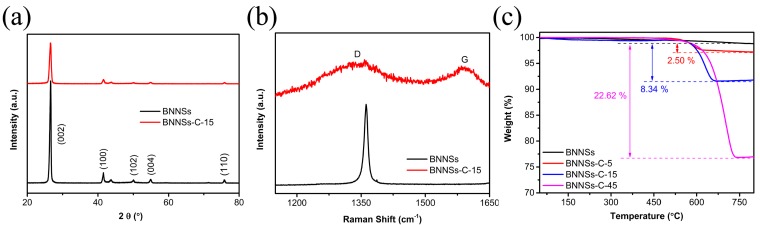
(**a**) XRD patterns; (**b**) Raman spectra; and (**c**) TGA curves of pristine BNNSs and corresponding BNNSs@C hybrids with different CVD deposition times.

The XRD patterns, Raman spectra, and TGA characterizations were conducted as exhibited in Figure 3 to obtain more precise structure and composition information of BNNSs@C hybrids. The XRD patterns of the pristine BNNSs and representative BNNSs@C-15 hybrids (Figure 3a) reveal that the peak intensity of BNNSs weakens after the deposition of carbon layer, which is attributed to the shielding effect of carbon layer. The existence of carbon layer was confirmed by Raman spectroscopy. As presented in Figure 3b, the BNNSs@C hybrids display two characteristic peaks around 1350 and 1580 cm^−1^, corresponding to the typical D and G bands of carbon species, respectively. The D band is usually attributed to the presence of amorphous or disordered carbon, while the G band is commonly associated with the *sp*^2^-bonded carbon atoms. As illustrated in Figure 3c, the TGA measurement was performed to calculate the mass fraction of carbon in the hybrids. For comparison, the TGA curve of pristine BNNSs is also displayed in Figure 3c. All three types of BNNSs@C hybrids exhibit a remarkable weight loss stage around 650 °C. Additionally, the mass fraction of carbon shell in the hybrids increases monotonously from 2.50 wt % for BNNSs@C-5 hybrids to 22.62 wt % for BNNSs@C-45 hybrids with increasing the CVD time from 5 to 45 min, which demonstrates that the composition of the hybrids could be precisely regulated by controlling the parameters involved in the CVD procedures.

**Figure 4:**
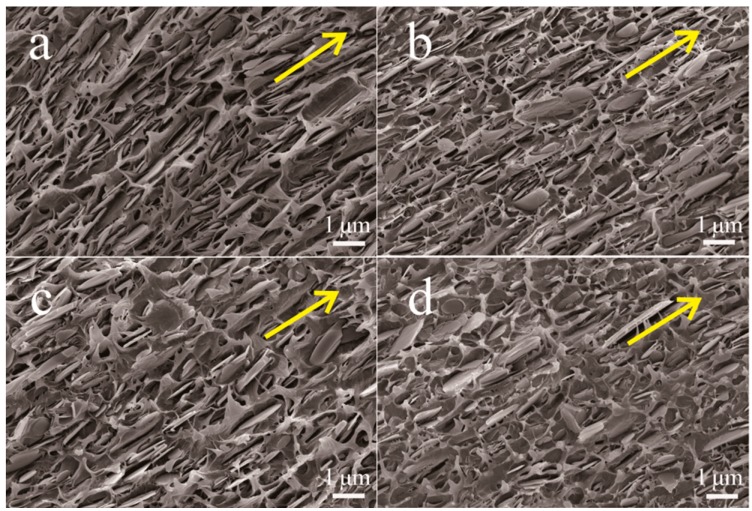
SEM images of the freeze-fractured cross-section surfaces of (**a**) BNNSs/PVDF; (**b**) BNNSs@C-5/PVDF; (**c**) BNNSs@C-15/PVDF; and (**d**) BNNSs@C-45/PVDF nanocomposites at 20 vol % filler loadings.

Figure 4 presents SEM images of the fracture surfaces of pristine BNNSs/PVDF and BNNSs@C/PVDF nanocomposites at 20 vol % filler loadings. We could observe that most parts of pristine BNNSs or BNNSs@C hybrids align well in the PVDF matrix with the direction of melting injection (as indicated by the yellow arrow in Figure 4) due to the applied shearing force from the injection process. The BNNSs@C hybrids are homogeneously distributed in the PVDF matrix. Moreover, the majority of BNNSs@C hybrids are buried well inside the polymer matrix because of their excellent interfacial bonding with the PVDF matrix. Additionally, the BNNSs@C hybrids are homogeneously distributed in the PVDF matrix. Such microstructure characteristics of freeze-fractured cross-section surfaces indicate that the BNNSs@C hybrids exhibit excellent compatibility with the PVDF matrix.

**Figure 5:**
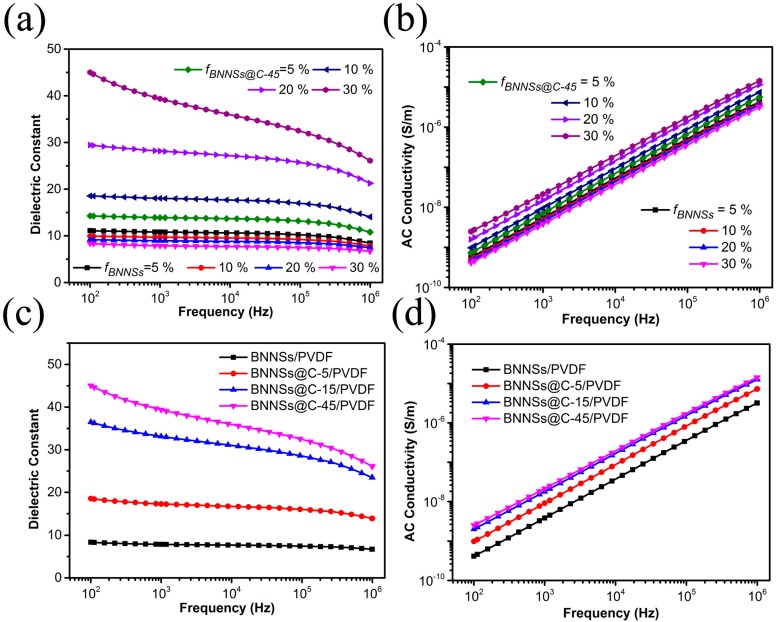
Frequency dependence of the (**a**) dielectric constant and (**b**) ac conductivity values of BNNSs/PVDF and BNNSs@C-45/PVDF nanocomposites at different filler loadings; Typical frequency-dependent comparison of (**c**) dielectric constant and (**d**) ac conductivity values of BNNSs/PVDF, BNNSs@C-5/PVDF, BNNSs@C-15/PVDF, and BNNSs@C-45/PVDF nanocomposites at equivalent filler loadings (30 vol %).

After demonstrating the successful deposition of carbon layer on the surface of BNNSs, the pristine BNNSs and BNNSs@C hybrids were incorporated into PVDF matrix separately by a combination of solution casting and melting blending methods. Figure 5 displays the frequency-dependent dielectric behaviors of the BNNSs/PVDF and BNNSs@C/PVDF nanocomposites incorporated with different types of BNNSs@C hybrids. As shown in Figure 5a, the BNNSs/PVDF nanocomposites exhibit weak frequency dependence characteristics and their dielectric constant decreases slightly with increasing *f_BNNSs_* up to 30 vol %. However, the frequency dependence of dielectric constant for BNNSs@C/PVDF nanocomposites at a low frequency range becomes more evident with increasing the *f_BNNSs@c_* up to 30 vol %. The strong frequency-dependent dielectric behavior could be attributed to the Maxwell-Wagner (MW) polarization, which occurs at the interfaces of heterogeneous components with different relaxation times [5]. The MW polarization only occurs at a low frequency range due to its long relaxation time. Therefore, the variation of dielectric constant and loss becomes relative steady at higher frequencies. The relaxation time (*τ*) is denoted by

(1) τ=ε/σ

where *ε* and *σ* represent the dielectric constant and conductivity, respectively. The relaxation time of BNNSs and PVDF matrix are several orders of magnitude larger than that of carbon layer. Thus, injected charges are tremendously blocked at the interfaces of BNNSs/carbon and carbon/PVDF, accordingly enhancing the dielectric constant at a low frequency range. Besides, the hybrid loading-dependent dielectric behavior of BNNSs@C/PVDF nanocomposites displays a totally different phenomenon compared with that of BNNSs/PVDF nanocomposites. The dielectric constant of BNNSs@C/PVDF nanocomposites increases significantly with increasing BNNS@C hybrid loading up to 30 vol %. For the part of ac conductivity, all BNNS/PVDF and BNNSs@C-45/PVDF nanocomposites exhibit similar frequency-dependent behavior. Their conductivity values show a strong dependence of frequency in the whole frequency range, which demonstrates their insulative characteristics. The conductivity values of BNNSs/PVDF nanocomposites decrease slightly with increasing the *f_BNNSs_* up to 30 vol %. Nevertheless, the conductivity values of BNNSs@C-45/PVDF nanocomposites increases with increasing the *f_BNNSs@C-45_* up to 30 vol %, which displays a similar law as discussed in the dielectric constant part. Figure 5c presents the comparison of frequency-dependent behavior for the BNNSs@C/PVDF nanocomposites incorporated with different types of BNNSs@C hybrids at an equivalent hybrids loading (30 vol %). The frequency dependence behavior of BNNSs@C/PVDF nanocomposites at a low frequency range becomes more significant by prolonging the CVD time in the carbon deposition procedure. Meanwhile, their dielectric constant increases significantly by prolonging the carbon deposition time from 5 to 45 min. Their conductivity values exhibit strong frequency dependent behavior in the whole frequency range, which reflects their insulative characteristics. Even if the nanocomposites are incorporated by the hybrids with a longer carbon deposition time (45 min) at a higher hybrids filler loading (30 vol %), it is still difficult to construct the percolation network in the PVDF matrix, which could be explained by the discontinuous carbon layer on the surface of BNNSs, as demonstrated in Figure 2. The naked surface of BNNSs inhibits the mobility of charge carrier, which directly prevents the construction of percolation network. Their conductivity values increase slightly by prolonging the carbon deposition time from 5 to 45 min.

**Figure 6:**
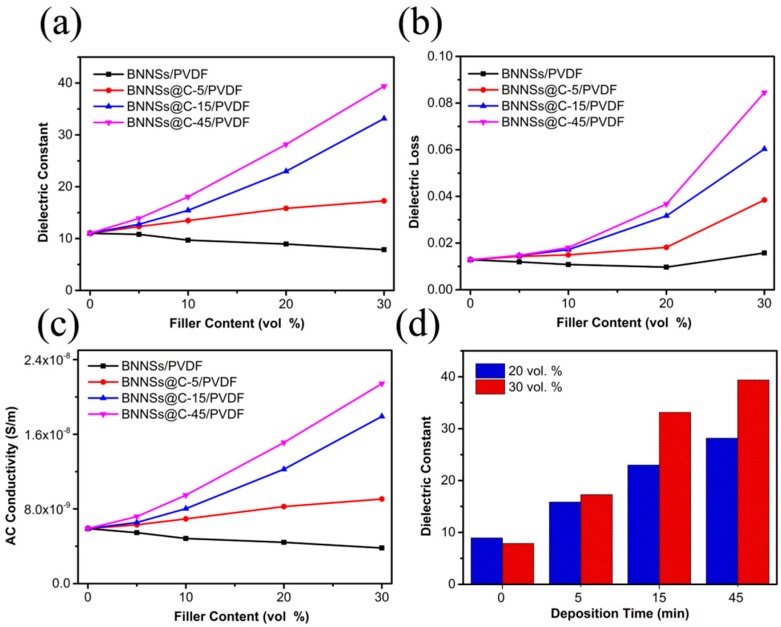
Dependence of the (**a**) dielectric constant; (**b**) dielectric loss; and (**c**) ac conductivity values of BNNSs/PVDF, BNNSs@C-5/PVDF, BNNSs@C-15/PVDF, and BNNSs@C-45/PVDF nanocomposites on the filler loadings (10^3^ Hz); (**d**) Typical variation of dielectric constant values (10^3^ Hz) of BNNSs/PVDF and BNNSs@C/PVDF nanocomposites as a function of carbon deposition time at different filler loadings (20 and 30 vol %).

Figure 6 presents the evolution of the dielectric properties of BNNSs/PVDF and BNNSs@C/PVDF nanocomposites with increasing the filler loadings. As shown in Figure 6a, the dielectric constant of BNNSs/PVDF nanocomposites decreases slightly from 11 to 8 with increasing the *f_BNNSs_* up to 30 vol %. However, the BNNSs@C/PVDF nanocomposites exhibit a monotonous increasing tendency with the increasing of hybrids loading. Typically, the dielectric constant increases from 11 to 39 for the BNNSs@C-45/PVDF nanocomposites with increasing the hybrids loading up to 30 vol %. The remarkable enhancement of dielectric constant after coating with the carbon layer could be mainly attributed to the gradual formation of the micro-capacitor network. Two types of micro-capacitors exist in BNNSs@C/PVDF nanocomposites. Each of BNNSs@C hybrid forms the first type of capacitor with the carbon layer as the electrode and BNNSs as the dielectric. The other kind of micro-capacitor consists of the adjacent conductive carbon layer in two different hybrids and the host PVDF in between, where the carbon layer and PVDF serve as electrodes and dielectric, respectively. These local micro-capacitor networks would be constructed with gradually increasing hybrids loading, which leads to the remarkable promotion of the dielectric permittivity. Besides, the dielectric constant of nanocomposites at the same amount of filler loading increases monotonously with the thickening carbon layer thickness of BNNSs@C hybrids. As illustrated in the Figure 6d, the dielectric constant augments from 8 for BNNSs/PVDF nanocomposites to 39 for BNNSs@C/PVDF nanocomposites at 30 vol % filler loading. The capacitance of parallel plate capacitor is given by

(2) $$C=\varepsilon_{0}\varepsilon_{eff}A/d$$

where *ε*_0_, *ε_eff_*, *A* and *d* represents the dielectric constant of vacuum, relative dielectric constant of nanocomposites, surface areas of the parallel plate, and the distance between two parallel plates, respectively. With prolonging the carbon deposition time, the elevated carbon content in the hybrids will undoubtedly thicken the carbon layer, resulting in dramatically reduction of PVDF dielectric thickness between the adjacent carbon layers. According to the Equation (2), the total capacitance stored in the second type of capacitor, as mentioned above, would sharply increase at the same amount of filler loading. Nevertheless, compared with the enhancement of dielectric constant in those percolative nanocomposites, the improvement of dielectric constant obtained in the current work is limited. The reported percolation behavior does not occur even if the nanocomposites is incorporated by the hybrids with a longer carbon deposition time (45 min) at a higher hybrids filler loading (30 vol %). The discontinuous carbon layer on the surface of BNNSs makes it difficult for the construction of percolation network. Therefore, the obtained enhancement of dielectric constant in current work is limited. As displayed in Figure 6b, the tendency of dielectric loss as a function of filler loading for BNNSs@C/PVDF nanocomposites exhibits the identical results that the loss increases monotonously with the increase of hybrid loading. For instance, the dielectric loss augments from 0.013 to 0.084 for the BNNSs@C-45/PVDF nanocomposites with increasing *f_BNNSs@C-45_* up to 30 vol %. Besides, the nanocomposites at the same amount of filler loading exhibit similar carbon layer thickness-dependent behavior of dielectric loss. The dielectric loss augments from 0.016 for BNNSs/PVDF nanocomposites to 0.084 for BNNSs@C-45/PVDF nanocomposites at an equivalent filler loading (30 vol %). The increased dielectric loss of the nanocomposites with increasing the carbon coating amount in the hybrids could be ascribed to thickening of the carbon layer around the surface of BNNSs, which shortens the distance for the adjacent BNNSs@C hybrids. Therefore, the resultant tunneling current is easier to pass through the adjacent hybrids, which leads to the increased dielectric loss after increasing the carbon coating amount. As for the conductivity part presented in Figure 6c, the filler loading and carbon layer thickness-dependent behavior of conductivity show the similar tendency that has been discussed in the dielectric constant and loss parts.

## 4. Conclusions

To conclude, the BNNSs@C hybrids with different carbon contents were synthesized by the CVD method. The carbon content in the as-obtained BNNSs@C hybrids could be accurately adjusted from 2.50 to 22.62 wt % through controlling the carbon deposition time. With the help of solution and melting blending methods, the BNNSs@C/PVDF nanocomposites were obtained by incorporating the BNNSs@C hybrids into PVDF matrix. The as-obtained BNNSs@C/PVDF nanocomposites exhibit an excellent dielectric performance and the dielectric properties could be accurately tuned by adjusting the carbon content. The improved interfacial polarizations of BNNSs/C and C/PVDF interfaces endow the nanocomposites with enhanced dielectric performance. These nanocomposites with distinguished dielectric performance could find their potential applications in the electronic industry.
